# Draft Genome Sequence of a Novel Lactate-Fermenting Bacterial Strain of the Family *Sporomusaceae* within the Class *Negativicutes*

**DOI:** 10.1128/MRA.01735-18

**Published:** 2019-03-07

**Authors:** Tomo Aoyagi, Yuta Kashiwabara, Hibiki Kurasawa, Seigo Amachi, Nobuyoshi Nakajima, Tomoyuki Hori, Shigeki Yamamura

**Affiliations:** a Environmental Management Research Institute, National Institute of Advanced Industrial Science and Technology (AIST), Tsukuba, Ibaraki, Japan; b Graduate School of Horticulture, Chiba University, Matsudo City, Chiba, Japan; c Center for Environmental Biology and Ecosystem Studies, National Institute for Environmental Studies, Tsukuba, Ibaraki, Japan; d Center for Regional Environmental Research, National Institute for Environmental Studies, Tsukuba, Ibaraki, Japan; University of Arizona

## Abstract

Here, we report a draft genome sequence of the *Sporomusaceae* bacterial strain FL31, a novel lactate-fermenting bacterium of the family *Sporomusaceae* within the class *Negativicutes*. This genome furthers our understanding of the physiological functions of this taxonomic group in natural environments.

## ANNOUNCEMENT

The class *Negativicutes* has recently been validated in the phylum *Firmicutes* and comprises 3 orders (1). The family *Sporomusaceae* of the order *Selenomonadales* has the largest number of genera, having 11 genera with 21 isolates (2). Members of this family have been found in various anoxic environments, including soils and subsurface sediments (3–5). However, little is known about the ecophysiological functions of this family. Genome information is important for the development of a thorough understanding regarding the metabolic potential of this taxonomic group. Here, we report a draft genome sequence of the lactate-fermenting *Sporomusaceae* bacterial strain FL31, isolated from an anoxic soil sample.

Strain FL31 was obtained from an anoxic soil sample (Matsudo City, Chiba, Japan [35°46'N, 139°53'E]) by using extinction dilution and a single-colony isolation technique. Strain FL31 was grown anaerobically in a minimum medium containing lactate (10 mM) as the sole carbon source. The medium contained the following ingredients (per liter): NH_4_Cl (0.535 g), KH_2_PO_4_ (0.136 g), MgCl_2_·6H_2_O (0.204 g), CaCl_2_·2H_2_O (0.147 g), trace mineral element solution (1 ml), vitamin solution (1 ml), Se/W solution (1 ml), and NaHCO_3_ (2.52 g). DNA was extracted by using a DNeasy blood and tissue kit (Qiagen, Germany). A paired-end DNA library (insert size, ∼550 bp) was generated using a TruSeq DNA PCR-free LT sample prep kit (Illumina, CA, USA). The library was sequenced with an Illumina MiSeq platform. The paired-end library generated 3,507,417 reads (300 bp paired end). Low-quality sequences (Q ≤ 10) were removed. Sequence assembly was performed by a *de novo* assembly using the CLC Genomic Workbench v. 11.0.1 (Qiagen) with default parameters, except for the minimum contig length (500 bp), and resulted in 118 scaffolds with 262.5× genome coverage from the library. The largest scaffold length was 821,961 bp. The *N*_50_ value was 264,930 with 118 scaffolds. The draft genome sequence of strain FL31 was 4,000,006 bp with a G+C content of 42.2%.

The BLASTn analysis of the 16S rRNA gene suggests that this strain is a member of a novel genus within the family *Sporomusaceae*. The closest phylogenetic cultured relatives of strain FL31 were Propionispora hippei KS^T^, “*Psychrosinus fermentans*” FCF9, and Pelosinus defluvii SHI-1^T^, with gene sequence identities of 93.5%, 93.2%, and 93.2%, respectively, in the nearly full length (1,552 bp) of the analyzed 16S rRNA gene sequences.

Annotation was carried out by using the DDBJ Fast Annotation and Submission Tool v. 1.0.0 (6) with default parameters. The draft genome sequence comprises 89 tRNA genes and 9 rRNA genes. Of the 3,821 predicted protein-coding sequences, 73.4% were assigned to recognized functional genes. The draft genome of strain FL31 contained the functional genes encoding l-lactate dehydrogenase, pyruvate dehydrogenase, malate dehydrogenase, fumarate hydratase, succinate dehydrogenase, acetate kinase, NADP-dependent malic enzyme, 2-oxo acid dehydrogenase, phosphate acetyltransferase, 4-hydroxybutyrate coenzyme A (CoA)-transferase, methylmalonyl-CoA carboxyltransferase, methylmalonyl-CoA epimerase, and methylmalonyl-CoA mutase, all of which are associated with lactate fermentation to propionate and acetate via the Wood-Werkman pathway (7).

The draft genome sequence of the *Sporomusaceae* bacterial strain FL31 provides insights into physiological functions of this family in anoxic natural environments.

### Data availability.

The *Sporomusaceae* bacterial strain FL31 draft genome sequences have been deposited as 118 scaffolds in DDBJ/EMBL/GenBank under accession numbers BIFV01000001 to BIFV01000118. The version described in this paper is the first version. The raw sequencing data have been deposited in the same database under the accession number DRA007976.
